# CytoSorb Therapy in COVID-19 (CTC) Patients Requiring Extracorporeal Membrane Oxygenation: A Multicenter, Retrospective Registry

**DOI:** 10.3389/fmed.2021.773461

**Published:** 2021-12-20

**Authors:** Tae Song, Jeremiah Hayanga, Lucian Durham, Lawrence Garrison, Paul McCarthy, Andy Barksdale, Deane Smith, Robert Bartlett, Mark Jaros, Peter Nelson, Zsolt Molnar, Efthymios Deliargyris, Nader Moazami

**Affiliations:** ^1^University of Chicago Medicine, Chicago, IL, United States; ^2^West Virginia University School of Medicine, Morgantown, WV, United States; ^3^Medical College of Wisconsin, Milwaukee, WI, United States; ^4^Franciscan Health Indianapolis, Indianapolis, IN, United States; ^5^New York University Grossman School of Medicine, New York, NY, United States; ^6^University of Michigan School of Medicine, Ann Arbor, MI, United States; ^7^Summit Analytical LLC, Denver, CO, United States; ^8^CytoSorbents Corporation, Princeton, NJ, United States; ^9^CytoSorbents Europe, Berlin, Germany; ^10^Department of Anesthesiology and Intensive Therapy, Semmelweis University, Budapest, Hungary; ^11^Department of Anesthesiology and Intensive Therapy, Poznan University of Medical Sciences, Poznan, Poland

**Keywords:** coronavirus–COVID-19, ECMO–extracorporeal membrane oxygenation, ICU–intensive care unit, mortality, inflammation, hemoperfusion, CytoSorb, hemoabsorption

## Abstract

**Introduction:** CytoSorb extracorporeal blood purification therapy received FDA Emergency Use Authorization (EUA) to suppress hyperinflammation in critically ill COVID-19 patients. The multicenter CTC Registry was established to systematically collect patient-level data, outcomes, and utilization patterns of CytoSorb under the EUA.

**Methods:** Patient-level data was entered retrospectively at participating centers. The primary outcome of the registry was ICU mortality. Patient disposition of death, continuing ICU care, or ICU discharge was analyzed up to Day 90 after start of CytoSorb therapy. Demographics, comorbidities, COVID-19 medications, inflammatory biomarkers, and details on CytoSorb use were compared between survivors and non-survivors in the veno-venous extracorporeal membrane oxygenation (ECMO) cohort.

**Results:** Between April 2020 and April 2021, 52 patients received veno-venous ECMO plus CytoSorb therapy at 5 U.S. centers. ICU mortality was 17.3% (9/52) on day 30, 26.9% (14/52) on day 90, and 30.8% (16/52) at final follow-up of 153 days. Survivors had a trend toward lower baseline D-Dimer levels (2.3 ± 2.5 vs. 19.8 ± 32.2 μg/mL, *p* = 0.056) compared to non-survivors. A logistic regression analysis suggested a borderline association between baseline D-Dimer levels and mortality with a 32% increase in the risk of death per 1 μg/mL increase (*p* = 0.055). CytoSorb was well-tolerated without any device-related adverse events reported.

**Conclusions:** CytoSorb therapy for critically ill COVID-19 patients on ECMO was associated with high survival rates suggesting potential therapeutic benefit. Elevated baseline D-Dimer levels may suggest increased risk of mortality. Prospective controlled studies are warranted to substantiate these results.

**Clinical Trial Registration:** https://clinicaltrials.gov/ct2/show/NCT0439192, identifier: NCT04391920.

## Introduction

From the outset of the Coronavirus Disease 2019 (COVID-19) pandemic there has been a rush to identify new adjunct therapies for critically ill COVID-19 patients to combat the high mortality observed in intensive care units (ICU) around the world (1). In the United States (U.S.) alone, mortality data from the Centers For Disease Control and Prevention (CDC) report that over 640,000 COVID-19 patients have died as of September 2021, mostly after admission to the ICU (2). To help address this unmet need, the U.S. Food and Drug Administration (FDA) has issued multiple Emergency Use Authorizations (EUA) since the start of the pandemic. CytoSorb therapy was granted EUA in April 2020 with the intent of treating hyperinflammation in adult COVID-19 ICU patients (3). The EUA was predicated on existing Conformité Européenne (CE) approval of CytoSorb in the European Union (E.U.) to remove inflammatory cytokines (4), and on clinical observations that hyperinflammation correlates with clinical decline and mortality in severe COVID-19 (5).

The CytoSorb device, a biocompatible adsorbent bead-filled cartridge, removes substances up to ~60 kilodaltons in size from blood, including a wide array of inflammatory mediators, by bead pore capture and surface binding of hydrophobic molecular moieties (4). CytoSorb can be incorporated in extracorporeal circuits including veno-venous extracorporeal membrane oxygenation (ECMO) platforms that are increasingly utilized globally to manage acute respiratory distress syndrome (ARDS) refractory to conventional mechanical ventilation in COVID-19 patients (Supplemental Figure 1) (3). Under the EUA, the intent of CytoSorb therapy for COVID-19 patients requiring ECMO is to reduce circulating inflammatory cytokines and other mediators that can cause ongoing injury to the lungs.

The Registry of CytoSorb Therapy in COVID-19 (CTC) ICU patients was launched shortly after the EUA to collect high-fidelity, patient-level data from U.S centers. Published case series and case reports to date on the use of CytoSorb in continuous renal replacement therapy (CRRT) or ECMO platforms have suggested potential clinical benefit in patients with COVID-19 requiring extracorporeal therapies [reviewed in (6)]. The CTC Registry is the first multicenter experience with CytoSorb therapy during ECMO in COVID-19 patients selected according to the EUA criteria (Supplemental Table 1). Here, we report on the primary outcome of the registry of ICU mortality for enrolled patients who received CytoSorb therapy *via* ECMO in the first year since issuance of the EUA. We also present a comparative exploratory analysis between ICU survivors and ICU non-survivors to determine if there are any discriminating patient characteristics or CytoSorb use between these groups that may reveal clues toward optimizing therapy under the EUA. The CTC Registry represents the first ever multicenter experience with CytoSorb therapy in COVID-19 patients and provides information that may help clinicians inform their provision of this novel adjunctive therapy.

## Methods

### U.S. FDA Emergency Use Authorization for CytoSorb Therapy

The clinical indication for CytoSorb (CytoSorbents Corporation, USA) therapy under the EUA is to hemoadsorb inflammatory cytokines in adult COVID-19 patients admitted to the ICU with imminent or confirmed respiratory failure as defined in the EUA (Supplemental Table 1). The CytoSorb device has neither been cleared nor approved by the FDA for the indication of treating patients with COVID-19. The EUA does not define exact criteria for hyperinflammation and therefore clinical decision making may be informed either by inflammatory marker levels or clinical evidence of “cytokine storm.” Accordingly, the decision to initiate CytoSorb therapy is at the discretion of the treating physician in the absence of any contraindications as provided in the EUA Instructions for Use (Supplemental Table 1) (3). The CytoSorb device can be easily integrated into extracorporeal circuits including those used for hemodialysis, hemoperfusion, CRRT, and ECMO. The integration of the CytoSorb device into a veno-venous ECMO circuit is depicted in Supplemental Figure 1. New providers of CytoSorb therapy under EUA undergo dedicated education and training on the EUA by the manufacturer (CytoSorbents Corporation) prior to device use. Systemic anticoagulation is provided using unfractionated heparin for standard anticoagulation targets for ECMO or continuous renal replacement therapy (CRRT). The EUA guidance for the duration of CytoSorb therapy permits at least 72 continuous hours with device exchange every 12 h in the first 24 h followed by device exchange every 24 h thereafter according to ongoing assessment of clinical benefit. Recommended blood flow rates through the CytoSorb device are 300–600 mL/min.

### CTC Registry Study Design

The CTC Registry (ClinicalTrials.gov Identifier: NCT04391920) is a multicenter observational study enrolling adult COVID-19 ICU patients treated with CytoSorb under the EUA. The registry does not enroll patients with COVID-19 who were not treated with CytoSorb therapy. All U.S. centers participating in the CTC Registry received approval from their respective Institutional Review Board for retrospective collection of de-identified patient-level data from medical records and entry into the CTC Registry electronic database. Consecutive patients from each participating center were entered without censoring to compile the first cohort of ECMO patients for analysis. Any device-related adverse events were required to be reported for review and determination of whether regulatory reporting was required under the EUA. The categories of collected data included demographics, comorbidities relevant to COVID-19 (7), COVID-19-related medications, inflammatory biomarkers (such as IL-6, D-dimers, ferritin, CRP), details on CytoSorb utilization and other extracorporeal therapies, and mortality. Data management was provided by a contract research organization (North America Science Associates). Patients who received CytoSorb therapy *via* ECMO in the year following issuance of the EUA (April 2020 to April 2021) were included in this study.

### Outcomes

The primary outcome was ICU mortality. Study follow-up determined both ICU survival and overall hospital survival. Patient follow-up was completed at the time of hospital discharge. A Kaplan-Meier time-to-event analysis of ICU survival was performed to depict the temporal distribution of death from the start of CytoSorb therapy (from Day 0 to Day 90). Patient disposition was also analyzed up to Day 90 according to three outcomes: death, continuing ICU care, or ICU discharge.

### Statistical Analysis

A pre-specified statistical analysis plan (Summit Analytical, LLC) was used to summarize and analyze the data variables. Available data were summarized using descriptive statistics. For continuous variables, the number of values (n) and the median, mean, standard deviation, minimum, and maximum were tabulated. For categorical variables, the counts and proportions of each value were tabulated. Change from baseline scores was calculated as the post-baseline measurement minus the baseline value or as percent of baseline. All analyses were performed using SAS v9.4.

An exploratory analysis was performed to investigate for differences in demographics, pre-existing comorbidities, COVID-19 medications, C-reactive protein (CRP), ferritin, D-Dimers, and CytoSorb utilization between ICU survivors and non-survivors. *T*-tests and Fisher's exact tests were performed for statistical comparison of continuous and categorical variables, respectively. A non-parametric Wilcoxon rank-sum test was used to compare baseline (pre-CytoSorb, Day 0) D-Dimer levels, due to non-normally distributed data. Paired *T*-tests were used to analyze the change in expression in CRP, ferritin, and D-Dimers from pre-CytoSorb (Day 0) to 72 h (Day 3) of CytoSorb therapy in patients with available paired laboratory values. A logistic regression analysis was used to investigate baseline D-Dimer levels as a predictor of ICU mortality.

## Results

### Patient Characteristics

Between April 2020 and April 2021, 52 ICU COVID-19 patients on ECMO were treated with CytoSorb therapy under EUA at 5 U.S. centers. They comprise the cohort for the current analysis, and by requiring ECMO, fulfilled the EUA clinical criteria of life-threating respiratory failure (i.e., criteria C.1 in Supplemental Table 1). Baseline patient characteristics of the cohort (Table 1) include the following: medium (IQR) age of 48 (43,55); 65% male; median (IQR) body mass index (kg/m^2^) of 34 (30,37); and pre-existing comorbidities for COVID-19 of obesity in 73%, hypertension in 40%, diabetes in 33%, and asthma in 17%. Details on COVID-19 specific therapies (i.e., the medications reported were hydroxychloroquine-azithromycin, lopinavir-ritonavir, remdesivir, dexamethasone, methylprednisolone, convalescent plasma, tocilizumab, and sarilumab) in the cohort included 67% receiving at least one anti-viral and/or immunomodulatory medication pre-ECMO and 46% of patients receiving at least one anti-viral and/or immunomodulatory medication during ECMO plus CytoSorb therapy. Treatment with dexamethasone, the only drug proven to date to lower ICU mortality in severe COVID-19 (8), occurred in 33 and 17% of the cohort pre-ECMO and during ECMO plus CytoSorb therapy, respectively. Baseline Sequential Organ Failure Assessment (SOFA) score in the cohort at the start of CytoSorb therapy was 6.9 ± 3.87.

**Table 1:** Baseline characteristics of the CTC Registry ECMO cohort.

**Baseline patient characteristics**		**CTC Registry** **ECMO cohort** **(*n* = 52)**
Demographics		
Sex:	Male	65% (34/52)
	Female	35% (18/52)
Age (years)		48 (43,55)
Race:	White	65% (34/52)
	Black	6% (3/52)
	Asian	4% (2/52)
	Other	6% (3/52)
	Not reported	19% (10/52)
BMI (kg/m^2^):		34 (30,37)
Pre-existing comorbidities		
Obesity (BMI ≥ 30 kg/m^2^)		73% (37/51)
Hypertension		40% (21/52)
Diabetes		33% (17/52)
Asthma		17% (9/52)

*Percent shown for categorical variables; Median (IQR) for continuous variables*.

### Provision of CytoSorb Therapy

Patient selection criteria and instructions for use for CytoSorb are outlined in the EUA, including a guidance for 72 h of therapy with device change every 12 h for the first 24 h followed by device change every 24 h thereafter. Each participating center received two dedicated training sessions prior to initiating treatment. First, a physician training video call to review patient selection criteria and device use instructions under the EUA, and second, an operational call with the responsible ECMO teams reviewing device integration and use instructions. Continuation of therapy after 72 h was allowed based on the potential for additional clinical benefit as determined by the treating physicians. In the cohort, CytoSorb therapy was initiated on average 7.0 ± 6.98 days after ICU admission, 3.6 ± 4.54 days after the start of mechanical ventilation (MV), 2.4 ± 6.02 days after the start of ECMO, and was continued for an average of 82.1 ± 30.13 h. Total ECMO time in the cohort was on average 31.6 ± 25.5 days. Of note, 24 of 52 patients were transfers from outside ICUs to the ICU of the participating center where ECMO plus CytoSorb therapy was provided. In these patients, duration of ICU stay and MV included the entire period from both hospitals. No unanticipated device-related adverse events were reported under the EUA for the duration of the registry.

The EUA indication for CytoSorb therapy is to suppress hyperinflammation in COVID-19 ICU patients. Two clinical inflammatory biomarkers, CRP and ferritin, were most often used by the participating centers to characterize the degree of inflammation in their COVID-19 patients and are listed in Table 2, showing that both markers were elevated in ICU survivors and non-survivors prior to CytoSorb therapy. Since the CTC Registry collects data derived from standard of care practices at each center, the type of laboratory tests performed, and the frequency of testing demonstrated substantial variability across participating centers. Notably, cytokines and procalcitonin were not routinely measured. For the purposes of identifying the impact of CytoSorb therapy on inflammatory markers, only patients in the cohort who had laboratory values on CRP and ferritin before the start (Day 0) and at 72 h (Day 3) of CytoSorb therapy were analyzed (Supplemental Table 2). CRP and ferritin decreased from 144 ± 189.1 mg/L to 98 ± 90.0 (*p* = 0.299, *n* = 22) and 1768.0 ± 1815.89 to 1314.8 ± 970.02 ng/mL (*p* = 0.260, *n* = 17), respectively. Comparison of D-Dimer levels pre-CytoSorb (Day 0) and at 72 h (Day 3) of CytoSorb therapy showed that D-Dimer levels were elevated above normal and did not decrease significantly with therapy (9.4 ± 23.46 to 7.1 ± 7.60 μg/mL, *p* = 0.658; *n* = 19).

**Table 2:** ICU mortality outcomes in the ECMO cohort.

**Parameter**	**CTC Registry ECMO cohort** (***n*** **= 52)**		
**ICU Mortality Overall**	30.8% (16/52)		
** 30-day ICU mortality**	17.3% (9/52)		
** 90-day ICU mortality**	26.9% (14/52)		
**Survivors (S) vs. Non-Survivors (NS)**	**S (*n* = 36)**	**NS (*n* = 16)**	***P*** **-value**
Demographics			
Sex:			
Male	67% (24/36)	63% (10/16)	0.764
Female	33% (12/36)	38% (6/16)	
Age (years)	47.4 ± 9.54	51.2 ± 7.38	0.161
Race:			
White	61% (22/36)	75% (12/16)	0.809
Black	8% (3/36)	0%	
Asian	3% (1/36)	6% (1/16)	
Other	6% (2/36)	6% (1/16)	
Not reported	22% (8/36)	13% (2/16)	
BMI (kg/m^2^)	33.1 ± 6.35	34.6 ± 6.95	0.460
Pre-existing comorbidities			
Obesity (BMI ≥ 30 kg/m^2^)	77% (27/35)	63% (10/16)	0.322
Hypertension	36% (13/36)	50% (8/16)	0.375
Diabetes	31% (11/36)	38% (6/16)	0.751
Asthma	19% (7/36)	13% (2/16)	0.704
COVID-19 medications			
At least one prior to ECMO	69% (25/36)	63% (10/16)	0.751
At least one during ECMO + CS	42% (15/36)	56% (9/16)	0.378
Dexamethasone prior to ECMO	33% (12/36)	31% (5/16)	0.999
Dexamethasone during ECMO + CS	11% (4/36)	31% (5/16)	0.113
SOFA score at start of CytoSorb therapy	6.3 ± 3.64 (*n* = 33)	8.3 ± 4.13 (*n* = 15)	0.104
CRP (mg/L) pre-CytoSorb (Day 0)	147 ± 185.1 (*n* = 23)	160 ± 106.3 (*n* = 6)	0.871
Ferritin (ng/mL) pre-CytoSorb (Day 0)	1311.0 ± 1073.88 (*n* = 19)	2282.5 ± 2834.91 (*n* = 6)	0.215
D-Dimer levels (μg/mL) pre-CytoSorb (Day 0)	2.3 ± 2.49 (*n* = 15)	19.8 ± 32.16 (*n* = 9)	0.056
MV indices and ABGs pre-CytoSorb (Day 0)			
Peak pressure (cm H_2_O)	28.5 ± 8.57 (*n* = 15)	32.0 ± 7.75 (*n* = 7)	0.365
Positive end expiratory pressure (cm H_2_O)	12.1 ± 3.30 (*n* = 16)	14.4 ± 4.16 (*n* = 7)	0.169
FiO_2_	0.76 ± 0.285 (*n* = 18)	0.95 ± 0.093 (*n* = 8)	0.016
PaO_2_ (mmHg)	73.3 ± 26.45 (*n* = 18)	57.4 ± 9.12 (*n* = 8)	0.033
PaCO_2_ (mmHg)	55.1 ± 16.13 (*n* = 18)	69.8 ± 33.10 (*n* = 8)	0.265
PaO_2_/FiO_2_ (mmHg)	118.8 ± 68.55 (*n* = 18)	60.8 ± 11.00 (*n* = 8)	0.003
Pharmacologic hemodynamic support pre-CytoSorb (Day 0)	28% (10/36)	56% (9/16)	0.055
CytoSorb therapy			
Duration of therapy (hours)	79.4 ± 27.72	88.3 ± 35.11	0.326
Time to therapy after ICU admission (days)	6.1 ± 7.40	9.1 ± 5.60	0.168
Time to therapy after MV start (days)	3.7 ± 4.83	3.2 ± 3.92	0.702
Time to therapy after ECMO start (days)	2.3 ± 6.63	2.8 ± 4.54	0.781
Total ECMO time (days)	29.1 ± 23.40	37.4 ± 29.71	0.282
Renal replacement therapy in ICU	17% (6/36)	31% (5/16)	0.281
Plasmapheresis therapy in ICU	3% (1/36)	13% (2/16)	0.221

*Percent shown for categorical variables; Mean ± standard deviation shown for continuous variables. P-values for categorical variable comparisons are from Fisher's exact tests; P-values for continuous variable comparisons are from independent samples T-tests. CS, CytoSorb therapy*.

### ICU Mortality

In the cohort, we observed an in hospital 90-day mortality rate of 26.9% and an in hospital mortality rate of 30.8% during maximal follow-up of 153 days (Table 2). The centers included in the registry had 90-day mortality rates ranging from ~10 to 50% of the total patients treated. ICU mortality for the 24 patients transferred from outside ICUs was 37.5% (9/24), whereas ICU mortality of the 28 patients that had not been transferred from outside ICUs was 25% (7/28). Comparisons of baseline patient characteristics, time to CytoSorb start after MV start or ECMO start, and CytoSorb use duration between the 36 ICU survivors and 16 ICU non-survivors did not identify any significant differences, although there were trends toward earlier initiation of CytoSorb therapy after ICU admission, lower baseline SOFA score, and lower rates of pharmacologic hemodynamic support pre-CytoSorb (Day 0) among the survivors (Table 2). Available data on MV parameters and arterial blood gases (ABGs) pre-CytoSorb (Day 0), when complete lung rest MV protocols including no MV during ECMO are often utilized, suggested that non-survivors had worse pulmonary function than survivors before CytoSorb therapy (e.g., PaO_2_/FiO_2_ of 60.8 ± 11.00 (*N* = 8) vs. 118.8 ± 68.55 mmHg (*N* = 18), *P* = 0.003, respectively), albeit the data are from only a fraction of the cohort. Use of COVID-19 medications pre-ECMO or during ECMO with CytoSorb therapy, and in particular dexamethasone, was also not significantly different between survivors and non-survivors.

Comparison of D-Dimer levels pre-CytoSorb (Day 0) between ICU survivors (*n* = 15) and non-survivors (*n* = 9), however, showed a nearly 10-fold difference in mean baseline D-Dimer levels between the groups: 2.3 ± 2.49 vs. 19.8 ± 32.16 μg/mL, *p* = 0.056, respectively. A logistic regression analysis to explore baseline D-Dimer level as a predictor of ICU mortality suggested a borderline association with a 32% increase in the risk of death per 1 μg/mL increase in D-Dimer levels [95% confidence interval: (0.994, 1.741), *p* = 0.055].

**Figure 1:**
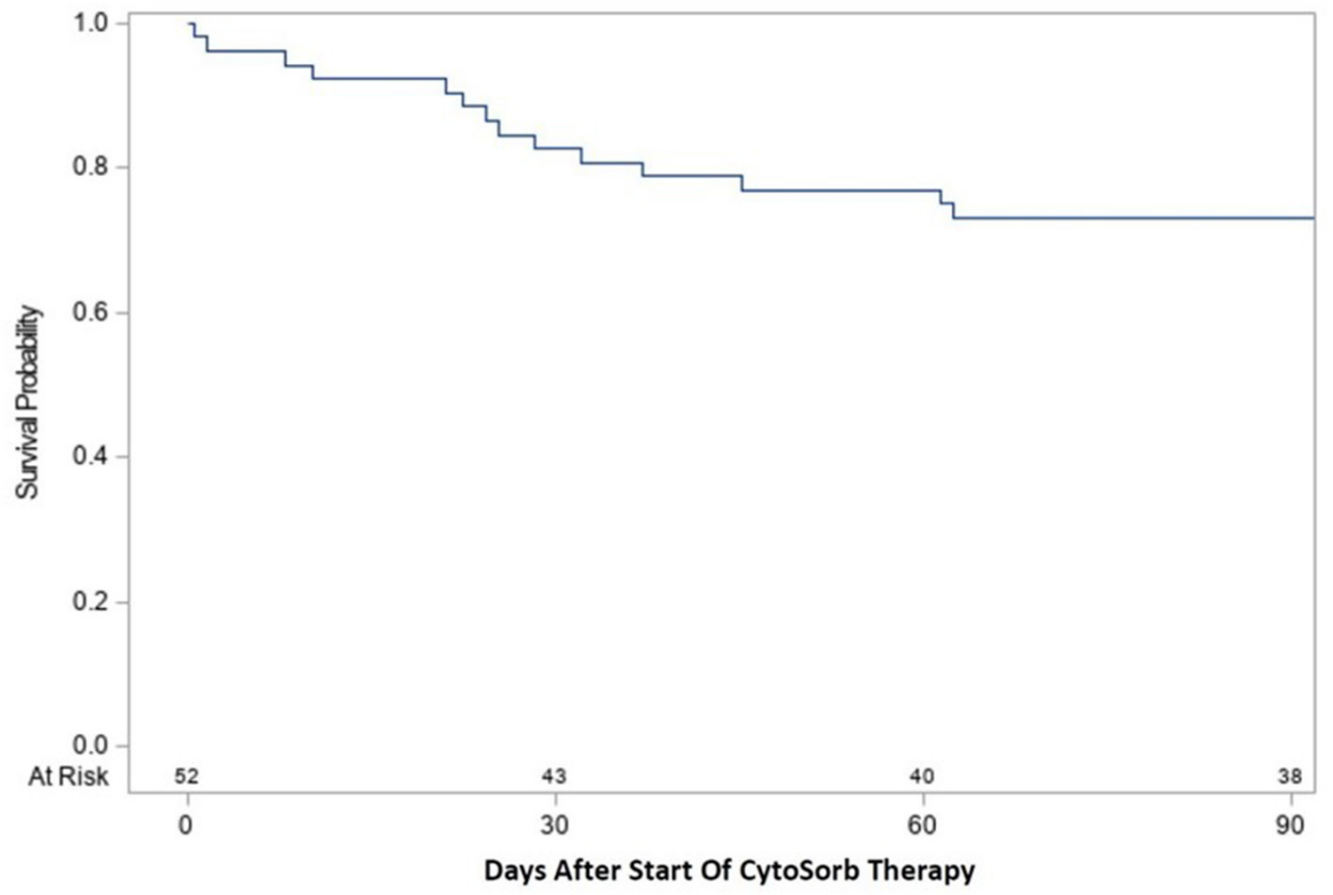
Kaplan-Meier curve for ICU survival from start of CytoSorb therapy to 90 days. Survival following start of CytoSorb therapy was 82.7% (43/52) at 30 days and 73.1% (38/52) at 90 days.

**Figure 2:**
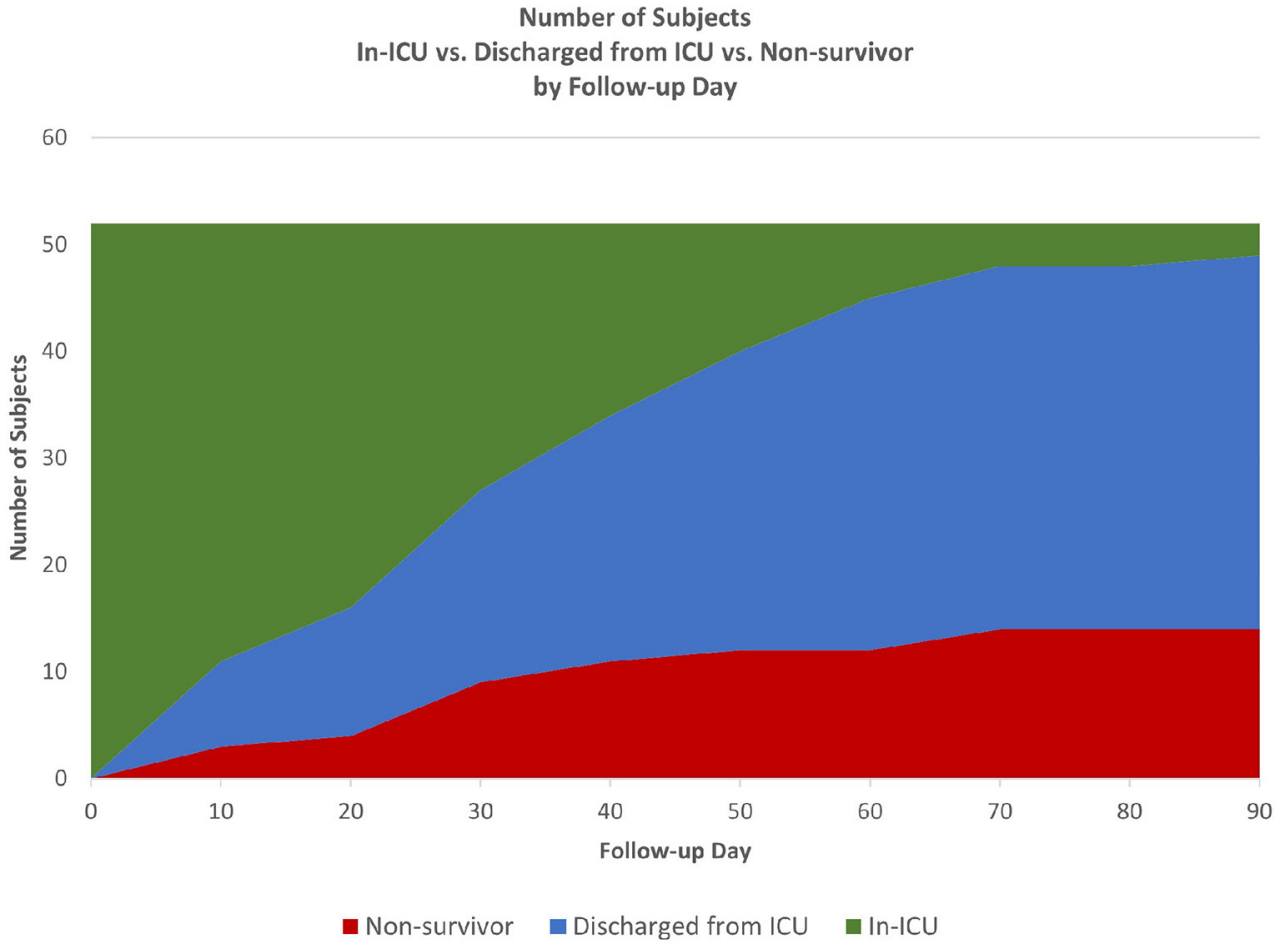
Patient disposition from start of CytoSorb therapy to 90 days. Stacked Area Graph showing the disposition of the CTC cohort following start of CytoSorb therapy in the ICU.

Time-to-event analysis (Figure 1) of the cohort for ICU survival after initiation of CytoSorb therapy showed 17.3% (9/52) died by Day 30 and 26.9% (14/52) died by Day 90, with the remaining 2 deaths in the ICU occurring after 90 days and the last patient discharged from the ICU at 153 days. Notably, all patients who were discharged from the ICU also survived to hospital discharge. Further characterization of the disposition of patients in the cohort is depicted by the Stacked Area Graph (Figure 2) showing that the rates of ICU discharge consistently exceeded the rates of ICU mortality in the 90 days following initiation of CytoSorb therapy. By Day 90, 67.3% (35/52) had been discharged from the ICU, 26.9% (14/52) had died in the ICU, and 5.8% (3/52) remained in the ICU.

## Discussion

The CTC Registry represents the first multicenter experience with the use of CytoSorb in critically ill COVID-19 ICU patients on veno-venous ECMO. Compared to patient populations included in previous reports of CytoSorb usage (6), the CTC ECMO cohort is a more uniform patient population, based on the selection of patients according to the inclusion criteria outlined in the EUA issued by the FDA. The observed mortality rate in the cohort, consisting entirely of ICU mortality, was 26.9% at 90 days, with rates of ICU discharge exceeding rates of ICU mortality after the start of CytoSorb therapy. There was a trend toward lower baseline SOFA scores in survivors, earlier initiation of CytoSorb therapy after ICU admission, and lower rates of pharmacologic hemodynamic support pre-CytoSorb as compared to non-survivors. Pre-CytoSorb MV indices, particularly PaO_2_/FiO_2_ ratios, appeared to be worse in a fraction of non-survivors vs. survivors with data available at that timepoint, suggesting that severity of ARDS before CytoSorb therapy should be explored further as a potential mortality risk factor. Of note, baseline D-Dimer levels were approximately 10 times higher in non-survivors as compared to survivors, an observation consistent with several published reports suggesting the prognostic significance of this biomarker in critically ill COVID-19 patients (9–11). One of the unique pathophysiological hallmarks of COVID-19 is the development of a thrombotic microangiopathy driven by endothelial damage to capillaries and arterioles. This process appears to translate to rising levels of circulating D-Dimers potentially making this marker a measure of disease severity. Therefore, the presence of very high D-Dimer levels may reflect an advanced process of extensive microangiopathy and thrombosis with broad tissue hypoxia and ischemic injury that is less likely to respond to cytokine removal. Participating centers in the CTC Registry reported that CytoSorb therapy incorporated into the ECMO circuit was generally well-tolerated without any unanticipated device-related adverse events. Further studies are needed to determine if the non-significant trends reported above help to identify patients that may benefit from CytoSorb therapy, and the best method of application of this therapy with ECMO under the EUA.

Prior published observational studies on CytoSorb therapy in COVID-19 patients are derived from single-center experiences (6). Alharthy et al. reported their CytoSorb experience with 50 COVID-19 patients with ARDS, who required CRRT including four who also required ECMO (12). Patients in this cohort had an average ICU stay of 20.7 days, and the mortality at 28 days after ICU discharge was 30%. In contrast, all patients in the CTC cohort required ECMO in the ICU for an average of 31.6 days, with 11 of 52 patients also requiring CRRT, reflecting a cohort with more multisystem disease compared to the above study.

Other contemporaneous multicenter registries in North America that also report on mortality rates in COVID-19 patients requiring ECMO provide some context for the U.S. multicenter CTC Registry ECMO cohort described here. The North American COVID-19 ARDS cohort (*N* > 3,800 patients as of September 2021) reported by the ELSO ECMO COVID-19 Registry dashboard (13) lists a 90-day in hospital mortality of 52%. This nearly two-fold higher mortality rate to that in the CTC cohort might reflect the ELSO Registry enrollment of “all-comer” COVID-19 patients requiring ECMO, whereas patients in the CTC cohort are more uniform by EUA clinical criteria. Likewise, a recent report from another U.S. multicenter, retrospective registry on 292 COVID-19 patients requiring ECMO showed a 90-day in hospital mortality of 42% (14).

Recently the single-center randomized study, Cytokine Adsorption in Severe COVID-19 Pneumonia Requiring Extracorporeal Membrane Oxygenation (CYCOV), reported a significantly higher mortality at the 30-Day study endpoint in a cohort of 17 patients treated with CytoSorb on ECMO compared to 17 patients treated with ECMO alone (i.e., 82 vs. 24%, respectively) (15). The 30-Day ICU mortality in the CTC cohort detailed in this report is 17.3%. Despite randomization in CYCOV, there were some disparities between the groups, with higher CRP, ferritin, and D-Dimer levels in the CytoSorb group. Also, in contrast to other studies which have demonstrated reduction in cytokine levels including IL-6, there was no difference in IL-6 levels between the groups.

The FDA EUA for CytoSorb therapy does not stipulate exact clinical criteria for hyperinflammation in COVID-19 ICU patients as a threshold for treating patients. Nevertheless, CRP and ferritin were markedly elevated in patients at the start of therapy, consistent with levels observed in aggregated data from multiple studies of severe COVID-19 correlating high inflammatory markers with worse outcomes (16), and the levels decreased with CytoSorb therapy. Other single-center case series and case reports of CytoSorb therapy for severe COVID-19, that also include use of the CytoSorb device in the CRRT platform, have similarly reported that CytoSorb therapy suppressed hyperinflammation in their patients (6).

### Strengths and Limitations

This is the first multicenter study and the largest observational cohort to date, of COVID-19 patients on ECMO treated with CytoSorb therapy. Nevertheless, it has obvious limitations. The CTC Registry does not enroll a comparator control group at participating centers to directly compare patient outcomes in the absence of CytoSorb therapy. Thus, definitive conclusions on whether treatment with CytoSorb results in clinical benefit cannot be made from the clinical observations presented here, since there are no data presented from comparator patients not treated with CytoSorb. Additionally, the overall natural history of changes in expression of inflammatory biomarkers covering the entire course of patients' illness to further characterize the mechanism of action of the CytoSorb device is not assessed in the registry. However, there were trends toward some patient characteristics, such as lower SOFA scores, lower baseline D-Dimer levels, and earlier CytoSorb use, that may be able to better predict and optimize patient benefit from adjunctive CytoSorb therapy under the EUA, and collection of more data will further inform and advance the understanding of CytoSorb therapy for COVID-19 patients.

## Conclusions

CytoSorb therapy for COVID-19 patients on ECMO under EUA was associated with mortality rates that may indicate potential therapeutic benefit, a possibility requiring comparison to patients not receiving CytoSorb therapy to substantiate. Elevated baseline D-Dimer levels may suggest increased risk of mortality, and less response to cytokine adsorption therapy. The CTC Registry continues to enroll patients and generate observational clinical data that will help guide CytoSorb therapy under EUA for COVID-19 patients. Further studies will be needed to explore the hypothesis-generating results from the CTC Registry study and to elucidate more precisely how cytokine adsorption therapy may play a role in altering the course of COVID-19.

## Data Availability Statement

The datasets presented in this article are not readily available because the data that support the findings of this study are retained by CytoSorbents Corporation, the sponsor of the CTC Registry. The data are available upon reasonable written request and with written permission of the CytoSorbents Corporation. Requests to access the datasets should be directed to Peter Nelson, pnelson@cytosorbents.com.

## Ethics Statement

The studies involving human participants were reviewed and approved by the respective Institutional Review Boards at the U.S. centers participating in the CTC Registry for retrospective collection of de-identified patient-level data from medical records and entry into the CTC Registry electronic database. Written informed consent for participation was not required for this study in accordance with the national legislation and the institutional requirements.

## Author Contributions

PN is the principal investigator for the CTC Registry for protocol, data analysis designs, and supervised the data entry into the registry. PN and MJ accessed and verified the data. TS, PN, ZM, and ED wrote the first draft of the manuscript. Statistical analyses were done by MJ of Summit Analytical. TS and NM enrolled the first ECMO patients into the registry. TS, JH, LD, LG, PM, AB, DS, and NM supported the acquisition of patient data into the registry at their participating medical centers. TS, NM, JH, and PN constitute the Executive Committee for the registry. All authors had access to the data, contributed to interpretation of the data, and read and approved the final manuscript.

## Funding

This work was supported by CytoSorbents Corporation.

## Conflict of Interest

This study received funding from the CytoSorbents Corporation. The funder had the following involvement with the study: MJ was employed by company Summit Analytical LLC. PN and ED are employees of the CytoSorbents Corporation (Princeton, NJ, USA). ZM is an employee of CytoSorbents Europe (Berlin, Germany). The remaining authors declare that the research was conducted in the absence of any commercial or financial relationships that could be construed as a potential conflict of interest.

## Publisher's Note

All claims expressed in this article are solely those of the authors and do not necessarily represent those of their affiliated organizations, or those of the publisher, the editors and the reviewers. Any product that may be evaluated in this article, or claim that may be made by its manufacturer, is not guaranteed or endorsed by the publisher.

## Abbreviations

ABG: arterial blood gases
ARDS: acute respiratory distress syndrome
CDC: Centers for Disease Control and Prevention
CE: Conformité
Européenne
CRRT: continuous renal replacement therapy
COVID-19: Coronavirus Disease 2019
CRP: C-reactive protein
CTC: CytoSorb Therapy in COVID-19
EUA: Emergency Use Authorization
E.U.: European Union
ECMO: extracorporeal membrane oxygenation
ELSO: Extracorporeal Life Support Organization
FDA: Food and Drug Administration
ICU: intensive care unit
IQR: interquartile range
MV: mechanical ventilation
SOFA: Sequential Organ Failure Assessment
U.S.: United States.
