# Supplementary material for: CytoSorb Therapy in COVID-19 (CTC) Patients Requiring Extracorporeal Membrane Oxygenation: A Multicenter, Retrospective Registry
Source: Front Med (Lausanne). 2021 Dec 20;8:773461. doi: 10.3389/fmed.2021.773461 (PMC8720923; doi:10.3389/fmed.2021.773461)
Supplement: Supplementary file 1 [file Data_Sheet_1.PDF]

Supplemental Table 1. Clinical criteria for CytoSorb therapy under FDA EUA [3]

**EUA definition for confirmed or imminent respiratory failure is any one of the following conditions:**<br><br>A) Early acute lung injury (ALI)/early acute respiratory distress syndrome (ARDS); or<br><br>B) Severe disease, defined as:<br>1) dyspnea,<br>2) respiratory frequency ≥ 30/min,<br>3) blood oxygen saturation ≤ 93%,<br>4) partial pressure of arterial oxygen to fraction of inspired oxygen ratio < 300, and/or<br>5) lung infiltrates > 50% within 24 to 48 hours; or<br><br>C) Life-threatening disease, defined as:<br>1) respiratory failure,<br>2) septic shock, and/or<br>3) multiple organ dysfunction or failure.
**Contraindications to CytoSorb therapy under the EUA:**<br><br>1) Patients with very low platelet counts (< 20,000/µL)<br>2) Any pre-existing contraindication to extracorporeal therapy<br>3) Known allergies to extracorporeal circuit components<br>4) History of heparin-induced thrombocytopenia<br>5) Acute sickle cell crisis<br>6) Morbid obesity with BMI ≥ 40 kg/m^2<br>7) Any pre-existing advanced medical disease with life-expectancy less than 1 month<br>8) Treatment deemed clinically futile<br>9) Pregnancy
