# Supplementary material for: CytoSorb Therapy in COVID-19 (CTC) Patients Requiring Extracorporeal Membrane Oxygenation: A Multicenter, Retrospective Registry
Source: Front Med (Lausanne). 2021 Dec 20;8:773461. doi: 10.3389/fmed.2021.773461 (PMC8720923; doi:10.3389/fmed.2021.773461)
Supplement: Supplementary file 2 [file Data_Sheet_2.PDF]

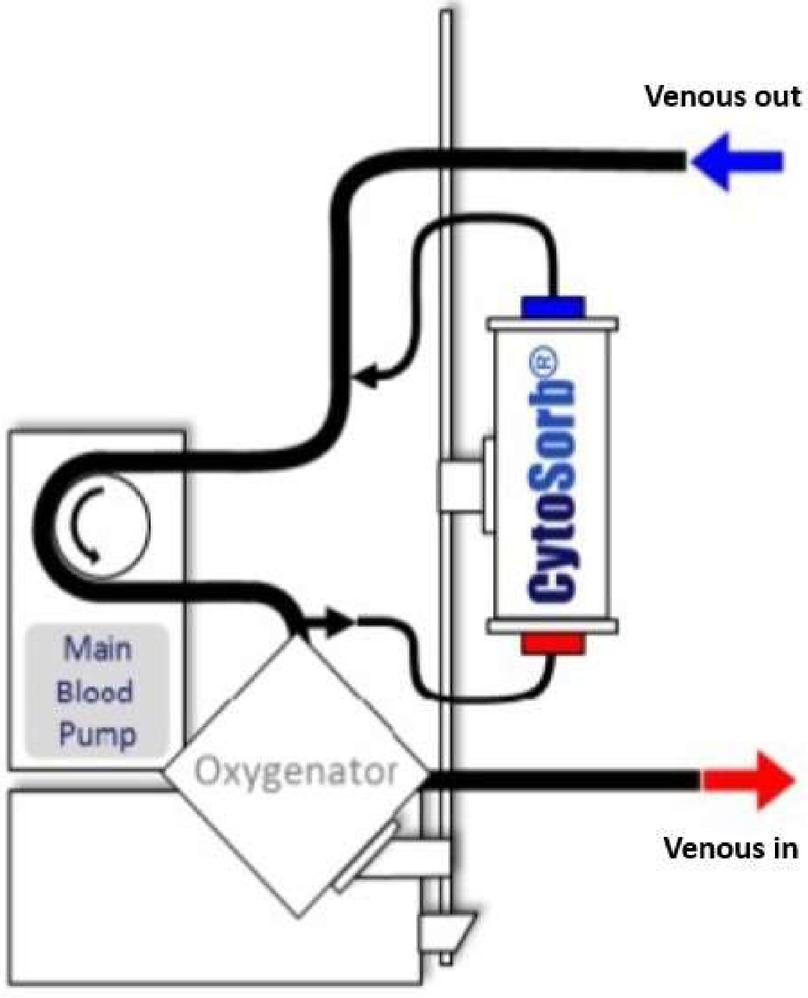

Supplemental Figure 1. Veno-venous ECMO platform with integration of the CytoSorb device.

The CytoSorb device is integrated as a shunt circuit in parallel to the main ECMO circuit. The red arrow indicates oxygenated blood from ECMO being returned to the patient.
