# Supplementary material for: CytoSorb Therapy in COVID-19 (CTC) Patients Requiring Extracorporeal Membrane Oxygenation: A Multicenter, Retrospective Registry
Source: Front Med (Lausanne). 2021 Dec 20;8:773461. doi: 10.3389/fmed.2021.773461 (PMC8720923; doi:10.3389/fmed.2021.773461)
Supplement: Supplementary file 3 [file Data_Sheet_3.PDF]

Supplemental Table 2. Expression of biomarkers with CytoSorb therapy

Biomarker*	Baseline – Day 0 (pre-CytoSorb)	Day 3 (72 hrs of CytoSorb)	Reduction**	P value
C-Reactive Protein (mg/L) (n=22)	144 ± 189.1	98 ± 90.0	32%	0.299
Ferritin (ng/mL) (n=17)	1768.0 ± 1815.89	1314.8 ± 970.02	26%	0.260
D-Dimers (µg/mL) (n=19)	9.4 ± 23.46	7.1 ± 7.60	24%	0.658

**Collected as part of standard of care. Only patients with both baseline and 72-hr samples included.*
***Biomarker reduction may be related to natural course of illness and/or CytoSorb-associated removal.*
